# Neurophobia among medical students and resident trainees in a tertiary comprehensive hospital in China

**DOI:** 10.1186/s12909-023-04812-1

**Published:** 2023-11-02

**Authors:** Fei Han, Yao Zhang, Ping Wang, Dong Wu, Li-Xin Zhou, Jun Ni

**Affiliations:** 1 Department of Neurology, Peking Union Medical College Hospital, Chinese Academy of Medical Sciences, Beijing, China; 2 Department of Respiratory Medicine, Peking Union Medical College Hospital, Chinese Academy of Medical Sciences, Beijing, 100730 China; 3 Department of Gastroenterology, Union Medical College Hospital, Chinese Academy of Medical Sciences, PekingBeijing, China

**Keywords:** Neurophobia, Medical students, Resident trainees, China

## Abstract

**Background:**

Medical students and residents have been revealed to have extraordinary difficulties in managing patients with neurological complaints. However, specific information on Chinese trainees is scarce. Herein, we aimed to uncover the presence of, contributing factors for, and potential solutions to neurophobia among medical students and resident trainees in China.

**Methods:**

Questionnaires were administered to the medical students of Peking Union Medical College and residents of the Internal Medicine Residency Training Program at Peking Union Medical College Hospital. We asked about perceived difficulty, knowledge, interest, and confidence in neurology in contrast to six other specialties. The reasons why neurology is regarded as difficult and approaches for improving neurological teaching have been appraised.

**Results:**

A total of 351 surveys were completed by 218 medical students and 133 residents. The response rate exceeded 70% in both groups. The prevalence of neurophobia was 66.1% and 58.6% among medical students and residents, respectively. Respondents declared that greater difficulty was observed in neurology than in other specialties, and the management of patients with neurological problems was the least comfortable (*p* < 0.0001). Neurophobia has various perceived causes, and neuroanatomy is regarded as the most important contributor. Nearly 80% of medical students felt that improvements in neurology teaching could be achieved through further integration of preclinical and clinical neurological teaching.

**Conclusions:**

The findings of the first survey on neurophobia in China are in accordance with those of previous studies. Neurophobia is highly prevalent in Chinese medical students and residents. Strategies to improve teaching, including enhanced integration of teaching and more online resources, are needed to prevent neurophobia.

**Supplementary Information:**

The online version contains supplementary material available at 10.1186/s12909-023-04812-1.

## Background

As the population ages, the burden of neurological diseases is intensifying [1]. Given the prevalence and influence of neurological disorders, more demand is placed on hospitals to deliver high-quality medical care. Therefore, it is critical for physicians to feel comfortable and capable of managing patients with neurological diseases.

Neurology is considered as a challenging component of the medical curriculum [2]. In 1994, the term neurophobia was proposed by Jozefowicz for describing ‘the fear of neuroscience and clinical neurology’ [3]. Since then, risk factors of and potential solutions for neurophobia among medical trainees and doctors in different countries have been investigated [4–8]. Based on these studies, neurophobia seems to be a global phenomenon that influences one-third to half of medical students and physicians at all stages of medical education. However, intervention measures in neurology education remain inadequate, and therefore, high-quality research is urgently needed to develop regimens to address this issue [9].

To date, no study has examined neurophobia in Chinese medical students or resident trainees. China's medical education system differs from those of the United States and European countries. Despite the disproportionate burden of neurological disorders in China, they are underrepresented in neurophobia literature. Further recognition of neurophobia in China is warranted to customize medical education programs, thereby recruiting future neurologists and giving non-neurologists confidence to deal with common neurological symptoms.

With this cross-sectional survey, we aimed to ascertain whether neurophobia exists among undergraduate medical students and resident trainees in a Chinese tertiary teaching hospital and, if so, why do they have difficulty in managing neurology? This study also investigated ways to prevent and overcome neurophobia by improving teaching. This is the first such study in the Chinese context.

## Methods

China has gradually established a clinical medical education system with Chinese characteristics and includes a 5-year and an 8-year program [10, 11]. The 5-year medical program forms the basis for clinical medicine education. The training plan includes basic and clinical medicine courses and internships. Undergraduates can continue their studies to earn a master’s or doctoral degree. The 8-year program aims to produce high-level innovative medical professionals. The 8-year program originated at Peking Union Medical College and has expanded to 14 colleges to date. The training plan includes basic science and clinical medical education, together with scientific research training. After graduation, most students obtain a Doctor of Medicine degree. Medical students in both programs must complete three years of standardized residency training after graduation to become qualified doctors.

This was a single-center study. We conducted a cross-sectional survey with two groups of respondents: undergraduate medical students and resident trainees in internal medicine. The first group included fifth- to eighth-year medical students from the Peking Union Medical College 8-year medical program. The second group comprised resident trainees who entered the standardized residency training program for internal medicine at Peking Union Medical College Hospital (PUMCH). The Ethics Board of PUMCH approved the study (reference number: K3821), and informed consent was obtained from all participants. All methods were performed in accordance with the relevant guidelines and regulations.

An online anonymous survey consisting of multiple-choice, 5-point Likert scale, and open-ended questions was re-designated from previous analogous studies (Supplementary material). The questionnaires were designed to appraise the degree of perceived difficulty, interest, knowledge, and confidence in neurology, compared to six other specialties (cardiology, gastroenterology, respiratory medicine, endocrinology, rheumatology, and nephrology). Furthermore, the questionnaire revealed possible contributors to neurophobia and probable strategies to improve neurology education for medical students and residents. All surveys were conducted in February 2023.

Based on previous studies [7], perceived difficulty and a lack of confidence are central features of neurophobia. Thus, neurophobia was defined as a combined confidence and difficulty score of less than or equal to 4 points. We also examined the presence of phobias in the six other medical specialties using a similar definition.

We analyzed the data as two independent datasets for undergraduate medical students and resident trainees. The data were processed using SPSS v24 for Windows statistical software package (SPSS Inc., Chicago, Illinois, United States). Replies to the Likert scale were tabulated into average scores. Values of *p* < 0.05 were considered significant. An independent t-test with the Bonferroni correction for multiple comparisons was used for comparison and significance.

## Results

A total of 351 surveys were completed (Table 1). The response rates for medical students and residents were 218/302 (72.2%) and 133/185 (71.9%), respectively, giving an overall response rate of 351/487 (72.1%). Among the medical students, 114 (52.3%) were female, and the mean age was 23.9 ± 1.3 years. For residents, 91 (68.9%) were female and the mean age was 27 ± 2.3 years.

**Table 1:** Demographic characteristics of the respondents

Medical students (*n* = 218)	
Age (years)	23.9 ± 1.3
Gender	
Male	104 (47.7%)
Female	114 (52.3%)
Subgroup	
5^th^ year medical student	60 (27.5%)
6^th^ year medical student	65 (29.8%)
7^th^ year medical student	52 (23.9%)
8^th^ year medical student	41 (18.8%)
Possibility of pursuing neurology as a future career	
Unlikely	86 (39.5%)
Moderate	67 (30.7%)
Likely	25 (11.5%)
Have not decided	40 (18.4%)
**Resident trainees (*n* = 133)**	
Age (years)	27 ± 2.3
Gender	
Male	42 (31.6%)
Female	91 (68.4%)
Subgroup	
Residents in the 1^st^ year	30 (22.6%)
Residents in the 2^nd^ year	34 (25.6%)
Residents in the 3^rd^ year	22 (16.5%)
Residents in the 4^th^ year	13 (9.8%)
Residents in the 5^th^ year	16 (12.0%)
Residents in the 6^th^ year and above	18 (13.5%)

### Neurophobia, difficulty, and confidence

**Fig. 1:**
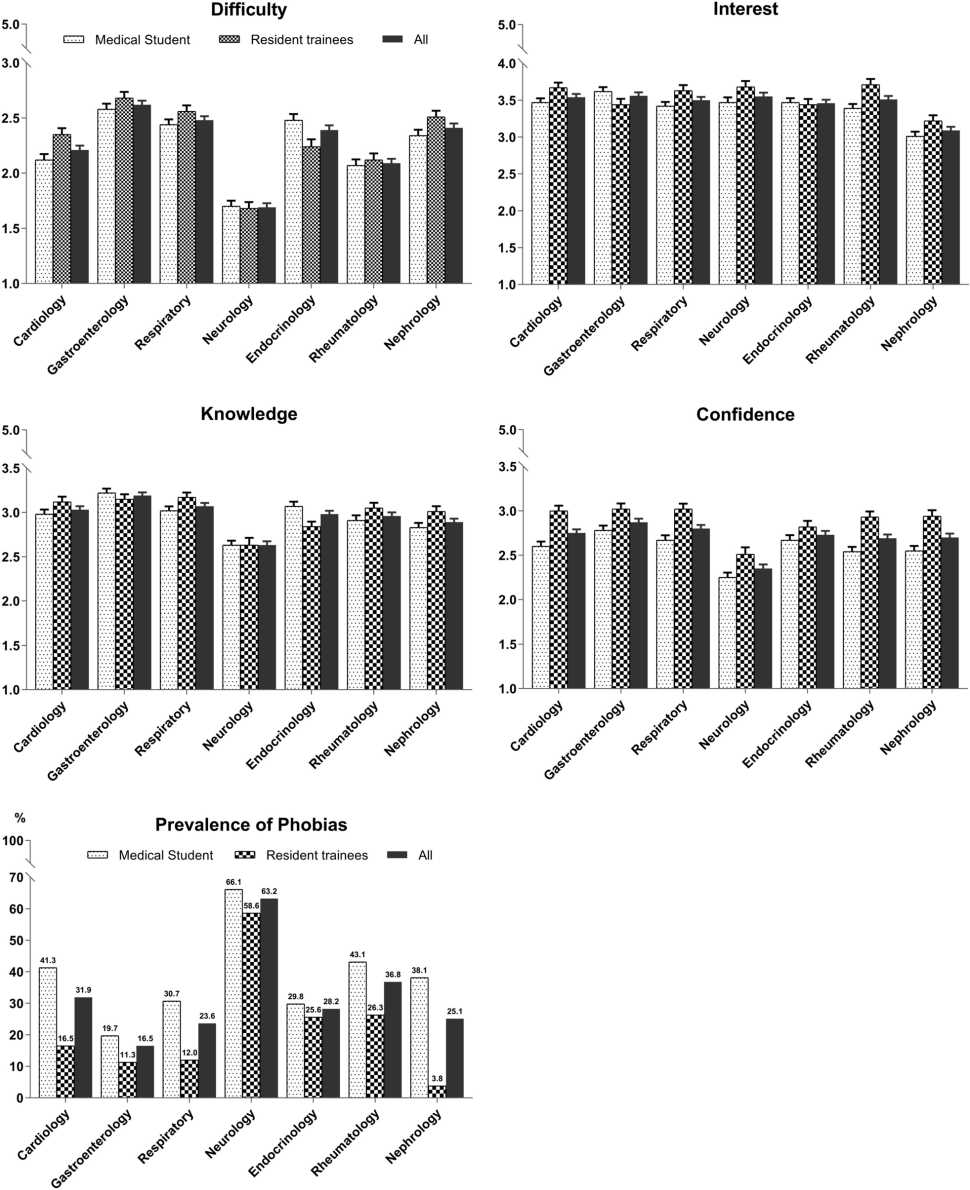
Mean survey score results and prevalence of phobias across six medical specialties. Difficulty ratings: 1 = very difficult, 2 = difficult, 3 = moderate, 4 = easy, 5 = very easy; Interest ratings: 1 = none, 2 = little, 3 = moderate, 4 = some, 5 = great; Knowledge ratings: 1 = very limited, 2 = limited, 3 = moderate, 4 = good, 5 = very good; Confidence ratings: 1 = very uneasy, 2 = uneasy, 3 = moderately confident, 4 = confident, 5 = very confident

According to our proposed definition, a higher prevalence of neurophobia was observed in medical students than in residents (144/218, 66.1% vs. 78/133, 58.6%; *p* = 0.007) (Fig. 1). The participants not only ranked neurology as the most difficult discipline but stated that it was far more difficult than the other six subjects (Table 2, *p* < 0.0001). This was equally clear among both medical students (difficulty score, mean ± standard error, 1.7 ± 0.05) and resident trainees (1.68 ± 0.06). Participants had minimal confidence in evaluating, diagnosing, and managing patients with neurological problems versus other medical conditions in both groups (confidence score 2.25 ± 0.05 for medical students and 2.51 ± 0.08 for resident trainees).

**Table 2:** Ratings of difficulty, interest, knowledge, and confidence for seven medical specialties

Specialty	Difficulty		Interest		Knowledge		Confidence	
	**Mean (SE)**	***p* value**	**Mean (SE)**	***p* value**	**Mean (SE)**	***p* value**	**Mean (SE)**	***p* value**
**Medical students (*n* = 218)**								
Cardiology	2.12 (0.05)	** < 0.0001**	3.47 (0.06)	0.96	2.98 (0.05)	** < 0.0001**	2.60 (0.05)	** < 0.0001**
Gastroenterology	2.58 (0.05)	** < 0.0001**	3.62 (0.06)	0.09	3.22 (0.05)	** < 0.0001**	2.78 (0.05)	** < 0.0001**
Respiratory	2.44 (0.05)	** < 0.0001**	3.42 (0.06)	0.54	3.02 (0.05)	** < 0.0001**	2.67 (0.05)	** < 0.0001**
Neurology	1.70 (0.05)	n/a	3.47 (0.07)	n/a	2.63 (0.05)	n/a	2.25 (0.05)	n/a
Endocrinology	2.48(0.06)	** < 0.0001**	3.47 (0.06)	0.99	3.07 (0.05)	** < 0.0001**	2.67 (0.06)	** < 0.0001**
Rheumatology	2.07 (0.05)	** < 0.0001**	3.39 (0.06)	0.34	2.91 (0.06)	**0.0003**	2.54 (0.05)	**0.0001**
Nephrology	2.34 (0.05)	** < 0.0001**	3.01 (0.06)	** < 0.0001**	2.83 (0.05)	**0.007**	2.55 (0.05)	** < 0.0001**
**Resident trainees (*n* = 133)**								
Cardiology	2.35 (0.06)	** < 0.0001**	3.67 (0.07)	0.94	3.12 (0.06)	** < 0.0001**	3.00 (0.06)	** < 0.0001**
Gastroenterology	2.68 (0.06)	** < 0.0001**	3.44 (0.08)	0.04	3.15 (0.06)	** < 0.0001**	3.02 (0.06)	** < 0.0001**
Respiratory	2.56 (0.05)	** < 0.0001**	3.63 (0.07)	0.68	3.17 (0.05)	** < 0.0001**	3.02 (0.06)	** < 0.0001**
Neurology	1.68 (0.06)	n/a	3.68 (0.08)	n/a	2.63 (0.08)	n/a	2.51 (0.08)	n/a
Endocrinology	2.24(0.07)	** < 0.0001**	3.44 (0.08)	0.04	2.84 (0.06)	0.034	2.82 (0.07)	**0.003**
Rheumatology	2.12 (0.06)	** < 0.0001**	3.71 (0.08)	0.79	3.05 (0.06)	** < 0.0001**	2.93 (0.06)	** < 0.0001**
Nephrology	2.51 (0.06)	** < 0.0001**	3.22 (0.08)	** < 0.0001**	3.01 (0.06)	**0.0002**	2.94 (0.07)	** < 0.0001**

Difficulty ratings: 1 = very difficult, 2 = difficult, 3 = moderate, 4 = easy, 5 = very easy

Interest ratings: 1 = none, 2 = little, 3 = moderate, 4 = some, 5 = great

Knowledge ratings: 1 = very limited, 2 = limited, 3 = moderate, 4 = good, 5 = very good

Confidence ratings: 1 = very uneasy, 2 = uneasy, 3 = moderately confident, 4 = confident, 5 = very confident

The significance threshold was adjusted for multiple comparisons using Bonferroni correction (*p* < 0.008 denotes significance). n/a, not applicable; SE, standard error

### Interest and knowledge

The gap in the level of interest between neurology and other medical specialties was small, except for nephrology, which had a significantly lower interest rating (*p* < 0.0001) (Table 2, Fig. 1). In contrast, a large gulf was observed in the knowledge scores (Table 2, Fig. 1). Respondents rated their neurology knowledge as the lowest among the seven medical specialties for medical students (2.63 ± 0.05). For residents, they also had significantly less knowledge in neurology (2.63 ± 0.08) compared to all other specialties, except for endocrinology.

### Factors for perceived difficulty with neurology

**Fig. 2:**
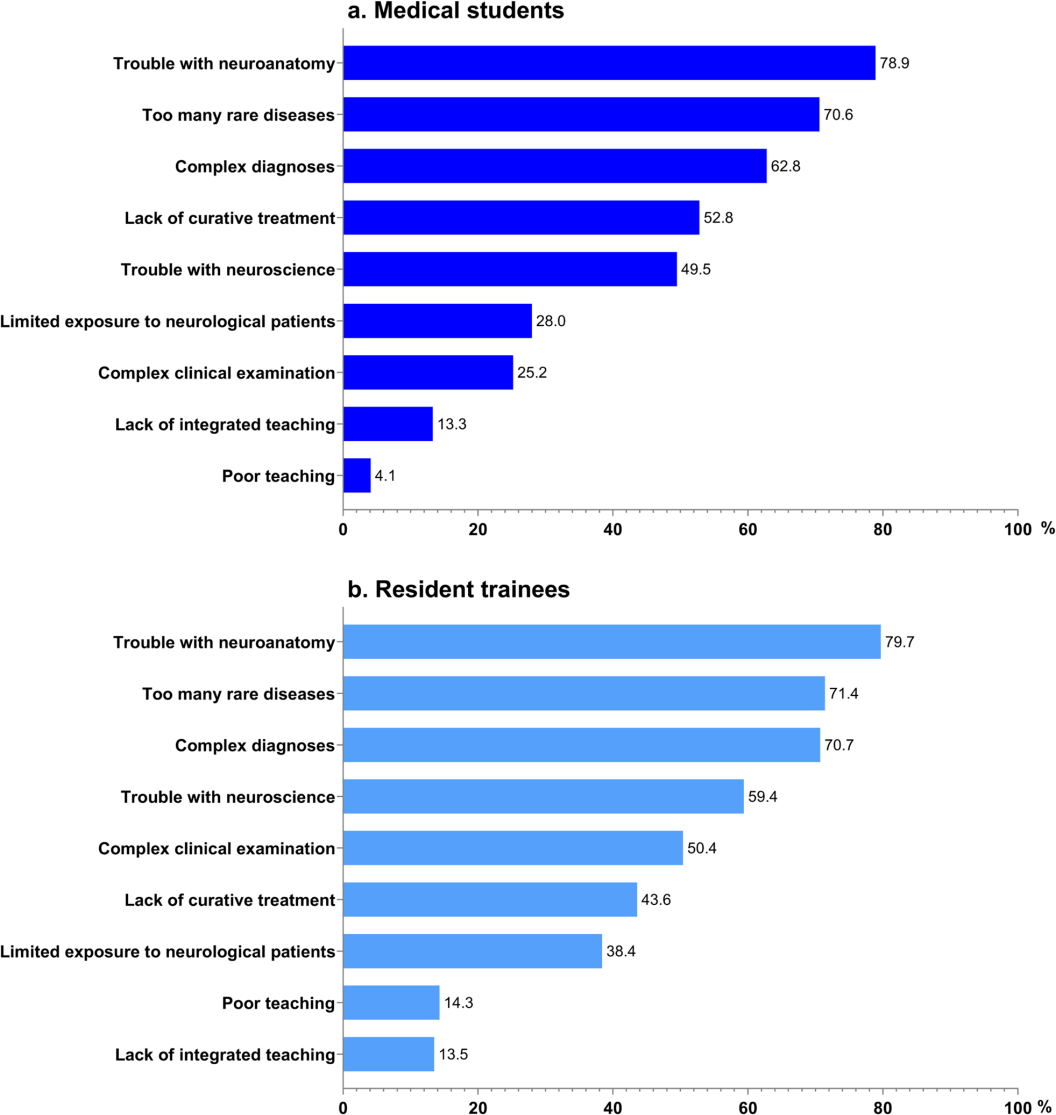
Reasons why medical students **a** and resident trainees **b** find neurology difficult

Various factors were associated with perceived difficulty in neurology (Fig. 2). Neuroanatomy was considered the most common factor contributing to making neurology difficult (78.9% for medical students; 79.7% for resident trainees), followed by a large number of rare disease diagnoses (70.6% for medical students; 71.4% for resident trainees) and diagnostic complexity (62.8% for medical students; 70.7% for resident trainees).

### Suggestions for improving neurology education

**Fig. 3:**
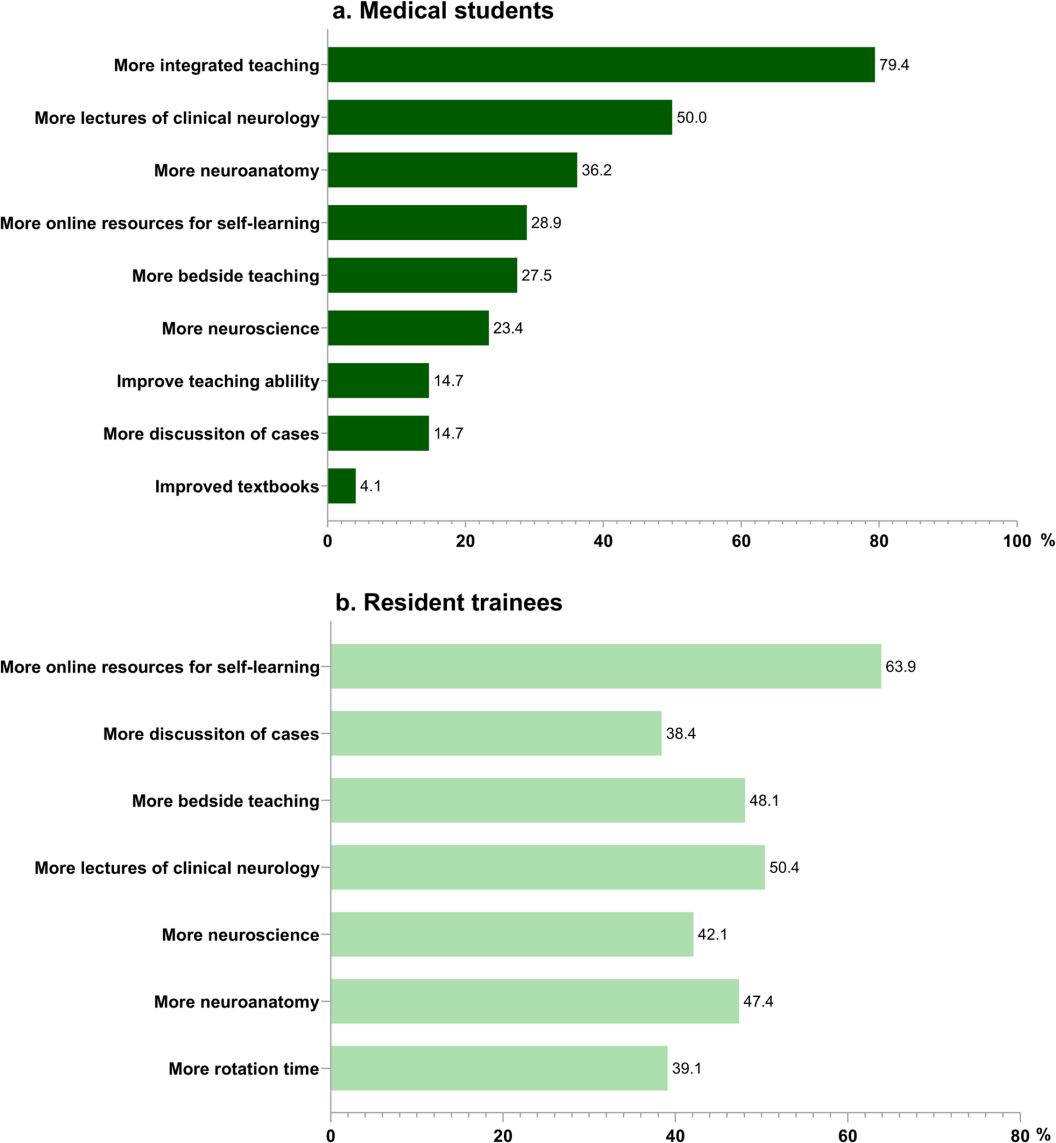
Percentage of respondents rating each approach to improve teaching. **a** medical students; **b** resident trainees

The responses indicated that approaches for improving neurology teaching for medical students consisted of more integrated teaching (79.4%), more lectures in clinical neurology (50%), and more effective neuroanatomy teaching (36.2%) (Fig. 3-a). In addition, residents mentioned having more online resources for self-directed learning (63.9%) and more bedside teaching during residency rotation (48.1%) (Fig. 3-b).

## Discussion

This study was the first structured survey of neurophobia among Chinese medical students and resident trainees, comprising 351 respondents from a tertiary teaching hospital in Beijing, China. Our results showed high difficulty and low confidence scores for neurology. This is in line with the results of prior studies in different parts of the world, including the United States, United Kingdom, Canada, South America, and Asian and African countries, revealing that neurophobia is a global issue across diverse educational systems [4–8, 12–15] (Table 3).

**Table 3:** Previous surveys on neurophobia

Country	Subjects	No	Difficulty	Three most common reasons of difficulty
United Kingdom [4]	medical students, senior house officers, general practitioner	345	Rank 1^st^ among the 7 medical specialties	Poor teaching Trouble with neuroanatomy Trouble with clinical examination
Ireland [5]	medical students and junior doctors	457	Rank 1^st^ among the 8 medical specialties	Limited patient exposure Complex diagnosis Not enough teaching
Trinidad & Tobago [16]	medical students	167	Rank 1^st^ among the 7 medical specialties	Need to know basic sciences Complex clinical examination Large number of diagnoses
United States [6]	medical students, residents	152	Rank 1^st^ among the 8 medical specialties	Limited patient exposure Neuroanatomy Insufficient teaching
Singapore [7]	medical students, junior doctors	289	Rank 1^st^ among the 7 medical specialties	-
Sri Lanka [14]	medical students, non-specialist doctors	248	Rank 1^st^ among the 7 medical specialties	1. Neuroanatomy 2. Complex clinical examination
United Kingdom [8]	medical students	2877	Rank 1^st^ among the 7 medical specialties	Neuroanatomy Basic neuroscience Lack of diagnostic certainty
Northern Ireland [12]	general practice trainees	118	Rank 1^st^ among the 7 medical specialties	Limited opportunity to work with a neurologist Not enough teaching Limited patient exposure
Canada [13]	medical students	187	24% afraid of clinical neurology 32% afraid of academic neuroscience	-
Saudi Arabia [17]	medical students, junior physicians	422	84.4% found neurology difficult	-
Fifteen countries in Africa [15]	medical students	294	Rank 1^st^ among the 7 medical specialties	Neuroanatomy Inadequate diagnostic tests Lack of diagnostic certainty

In this study, both medical students and residents agreed that neurology was the most difficult medical discipline, and they felt the least confident in dealing with patients with neurological problems, in contrast to the six other specialties in primary care settings. Two-thirds of the medical students and more than half of the resident trainees had neurophobia. This prevalence is higher than previous estimates by Jozefowcz [3] and a survey conducted in Singapore [7], indicating that neurophobia should be taken seriously in China. Over the past 30 years, neurology perception has remained unchanged in contrast to the rapidly changing requirements for neurological care in an aging population. Medical education authorities and neurology educators should pay particular attention to these issues.

Consistent with previous studies [4, 6, 8], neuroanatomy was the main reason for difficulty in neurology. In the digital era, neuroanatomy education can be improved from conventional sectional images by employing innovative strategies, such as computer-based instructional 3-dimensional models, web-based neuroscience and neurology teaching videos, blended and flipped strategies, and problem-based effective teaching in neuroanatomy.

The poor integration of preclinical and clinical neurological teaching is another major complaint. Almost 80% of the medical students stated that a combination of neuroanatomy, neuroscience, and clinical neurology would be the best approach. Fragmentation in the learning of basic neuroscience with clinical neurology should be tackled by integrating basic neuroscience learning with early, effective, and multiple clinical exposures more efficiently under a neuro-mentorship program. Furthermore, introducing preclinical revision courses in areas such as neuroscience and neuroanatomy through case-based learning when students enter clinical training could be another useful approach.

In Peking Union Medical College, medical students are required to be involved in a total of 8 weeks neurology attachment in the clerkship year (6^th^ year) and internship year (7^th^ year). The internal medicine residency training program included a 4-week rotation in the Department of Neurology at PUMCH. Some respondents suggested that the lack of rotation time and restricted exposure to neurological patients led them to consider neurology a difficult subject, which should be addressed urgently. In such a limited rotation time, multiple novel educational interventions would help students organize, re-engage, and manage their learning approaches for a deeper understanding through self‑directed, problem-based, and team-based learning.

In our study, a high proportion of the residents expected more online self-directed learning resources. Utilization of online resources in neurology teaching and its distinct success over other teaching approaches has been signified in prior studies [18–21]. Online teaching has been revealed to enhance neurology knowledge at the final clinical attachment and residency rotation stages compared to textbooks. The incorporation of video tutorials as part of the online educational approach could offer a reasonable addition to increasing patient exposure and bedside teaching for residents.

It is noteworthy that neurology is regarded as a difficult and challenging subject, but this did not reduce students’ interest in or enthusiasm for neurology, and a substantial number of medical students tended to pursue neurology in their future careers. However, once resident trainees begin clinical practice, they may become less neurophobic. Although there was a relatively wide range of neurophobias among medical students and young residents, a trend toward gradual improvement was observed. We speculate that ongoing neurological education and clinical exposure to overcome neurophobia will initially target medical students and then seamlessly continue via postgraduate education.

Owing to the unique, difficult, and complex nature of neurology, neurophobia has long existed worldwide, and our research reached the same conclusions. The presence of neurophobia in various medical communities around the globe raises concerns about its adverse effects on the quality of patient care and management. Researchers have presented several evidence-based recommendations for overcoming neurophobia. Neurology education curriculum reforms, a paradigm shift from a traditional knowledge-based curriculum to a student-centered, and competency-driven education [22], neuro-mentorship programs, evidence-based effective educational interventions, and problem-based and integrated learning, would be the way forward to removing neurophobia.

As China continues to grow, the need for physicians to adequately address the health needs of its population has become increasingly important. In the future, the government should provide more political support and financial investments to improve the overall capability of global cooperation and communication in neurology education, reinforce partnerships and cultures, identify differences between China and the rest of the world, propose targeted improvement measures to solve neurophobia, and ultimately provide excellent talent reserves for brain science in the twenty-first century.

This study had several limitations. This study was conducted in a single medical institution. PUMCH is a tertiary comprehensive teaching hospital in China and a national referral center offering diagnostic and therapeutic care for complex and rare disorders. Therefore, it may be difficult to generalize our findings to other Chinese medical schools and hospitals. Therefore, multi-center studies are required to confirm these conclusions. Investigations are also warranted to estimate whether intervention measures such as increased patient exposure, more online resources, and enhanced integration of neuroanatomy, neuroscience, and clinical neurology may result in better performance in neurology education.

## Conclusions

Neurophobia is prevalent, and both medical students and resident trainees consider neurology to be the most difficult specialty in Chinese tertiary comprehensive hospitals. This study sheds light on the factors that contribute to neurophobia and possible preventive approaches, which will be an essential step in training clinicians to cope with the growing challenges of managing patients with neurological disorders.

### Supplementary Information

**Additional file 1.**

## Data Availability

The datasets used and/or analyzed during the current study are available from the corresponding author on reasonable request.

## Abbreviation

PUMCH: Peking Union Medical College Hospital
