# Supplementary material for: Neurophobia among medical students and resident trainees in a tertiary comprehensive hospital in China
Source: BMC Med Educ. 2023 Nov 2;23:824. doi: 10.1186/s12909-023-04812-1 (PMC10621143; doi:10.1186/s12909-023-04812-1)
Supplement: Supplementary file 1 — Additional file 1. [file 12909_2023_4812_MOESM1_ESM.docx]

**Survey of medical students on the perception of Neurology**

1. **What is your age?**

________________

1. **What is your gender?**

| Male | Female |
| --- | --- |

1. **Which is your grade of the medical course?**

| 5^th^ year | 6^th^ year | 7^th^ year | 8^th^ year |
| --- | --- | --- | --- |

1. **How difficult do you think it is to learn about the following areas of medicine?**

|  | 1  Very difficult | 2  Difficult | 3  Moderate | 4  Easy | 5  Very easy |
| --- | --- | --- | --- | --- | --- |
| Cardiology |  |  |  |  |  |
| Respiratory medicine |  |  |  |  |  |
| Gastroenterology |  |  |  |  |  |
| Neurology |  |  |  |  |  |
| Endocrinology |  |  |  |  |  |
| Rheumatology |  |  |  |  |  |
| Nephrology |  |  |  |  |  |

1. **What is your current level of interest in the following areas of medicine?**

|  | 1 None | 2 Little | 3 Moderate | 4 Some | 5 Great |
| --- | --- | --- | --- | --- | --- |
| Cardiology |  |  |  |  |  |
| Respiratory medicine |  |  |  |  |  |
| Gastroenterology |  |  |  |  |  |
| Neurology |  |  |  |  |  |
| Endocrinology |  |  |  |  |  |
| Rheumatology |  |  |  |  |  |
| Nephrology |  |  |  |  |  |

1. **What is your current level of knowledge in the following areas of medicine?**

|  | 1 Little | 2 Some | 3 Moderate | 4 Fair | 5 Great |
| --- | --- | --- | --- | --- | --- |
| Cardiology |  |  |  |  |  |
| Respiratory medicine |  |  |  |  |  |
| Gastroenterology |  |  |  |  |  |
| Neurology |  |  |  |  |  |
| Endocrinology |  |  |  |  |  |
| Rheumatology |  |  |  |  |  |
| Nephrology |  |  |  |  |  |

1. **When you see a patient in your clinical experience with a complaint in the following area of Neurology, what do you feel?**

|  | 1  Very uneasy | 2  Uneasy | 3  Averagely competent | 4  Confident | 5  Very confident |
| --- | --- | --- | --- | --- | --- |
| Cardiology |  |  |  |  |  |
| Respiratory medicine |  |  |  |  |  |
| Gastroenterology |  |  |  |  |  |
| Neurology |  |  |  |  |  |
| Endocrinology |  |  |  |  |  |
| Rheumatology |  |  |  |  |  |
| Nephrology |  |  |  |  |  |

1. **Why is neurology difficult?**

| Trouble with neuroanatomy |
| --- |
| Trouble with basic neuroscience |
| Trouble with clinical examination |
| Trouble with diagnoses |
| Too many rare diagnoses |
| Lack of curative treatment in most instances |
| Poor teaching |
| Lack of integrated teaching |
| Limited exposure to neurological patients |
| Other comments (please specify) _______________ |

1. **How can teaching improved?**

| More integrated teaching (neuroscience-neuroanatomy-pathophysiology-disease) |
| --- |
| Improved textbooks |
| More neuroanatomy |
| More neuroscience |
| More lectures of clinical neurology |
| More bedside teaching |
| More discussion of cases |
| Improve teachers' teaching ability |
| More online self-learning resources |
| Other comments (please specify) _______________ |

**10. How likely are you to pursue a career in neurology?**

| Unlikely | Neither likely nor unlikely | Likely | Don’t know |
| --- | --- | --- | --- |

**Thank you for taking part in this survey. We welcome any other comments you may have on your perception and experience of neurology and how this can be improved.**

___________________________________________________________

**Survey of resident trainees on the perception of Neurology**

1. **What is your age?**

________________

1. **What is your gender?**

| Male | Female |
| --- | --- |

1. **What is your current year of residency?**

| 1^st^ year | 2^nd^ year | 3^rd^ year | 4^th^ year | 5^th^ year |
| --- | --- | --- | --- | --- |

1. **How difficult do you think it is to learn about the following areas of medicine?**

|  | 1  Very difficult | | 2  Difficult | | 3  Moderate | | 4  Easy | | 5  Very easy |
| --- | --- | --- | --- | --- | --- | --- | --- | --- | --- |
| Cardiology |  |  | |  | |  | |  | |
| Respiratory medicine |  |  | |  | |  | |  | |
| Gastroenterology |  |  | |  | |  | |  | |
| Neurology |  |  | |  | |  | |  | |
| Endocrinology |  |  | |  | |  | |  | |
| Rheumatology |  |  | |  | |  | |  | |
| Nephrology |  |  | |  | |  | |  | |

1. **What is your current level of interest in the following areas of medicine?**

|  | 1 None | 2 Little | 3 Moderate | 4 Some | 5 Great |
| --- | --- | --- | --- | --- | --- |
| Cardiology |  |  |  |  |  |
| Respiratory medicine |  |  |  |  |  |
| Gastroenterology |  |  |  |  |  |
| Neurology |  |  |  |  |  |
| Endocrinology |  |  |  |  |  |
| Rheumatology |  |  |  |  |  |
| Nephrology |  |  |  |  |  |

1. **What is your current level of knowledge in the following areas of medicine?**

|  | 1 Little | 2 Some | 3 Moderate | 4 Fair | 5 Great |
| --- | --- | --- | --- | --- | --- |
| Cardiology |  |  |  |  |  |
| Respiratory medicine |  |  |  |  |  |
| Gastroenterology |  |  |  |  |  |
| Neurology |  |  |  |  |  |
| Endocrinology |  |  |  |  |  |
| Rheumatology |  |  |  |  |  |
| Nephrology |  |  |  |  |  |

1. **When you see a patient in your clinical experience with a complaint in the following area of Neurology, what do you feel?**

|  | 1  Very uneasy | 2  Uneasy | 3  Averagely competent | 4  Confident | 5  Very confident |
| --- | --- | --- | --- | --- | --- |
| Cardiology |  |  |  |  |  |
| Respiratory medicine |  |  |  |  |  |
| Gastroenterology |  |  |  |  |  |
| Neurology |  |  |  |  |  |
| Endocrinology |  |  |  |  |  |
| Rheumatology |  |  |  |  |  |
| Nephrology |  |  |  |  |  |

1. **Why is neurology difficult?**

| Trouble with neuroanatomy |
| --- |
| Trouble with basic neuroscience |
| Trouble with clinical examination |
| Trouble with diagnoses |
| Too many rare diagnoses |
| Lack of curative treatment in most instances |
| Poor teaching |
| Lack of integrated teaching |
| Limited exposure to neurological patients |
| Other comments (please specify) _______________ |

1. **How would you rate the quality of the teaching you have received in the following period?**

|  | 1 Very poor | 2 Poor | 3 Moderate | 4 Good | 5 Very good |
| --- | --- | --- | --- | --- | --- |
| Medical students |  |  |  |  |  |
| Residency |  |  |  |  |  |

1. **How can teaching improved?**

| More time of residency rotation in neurology |
| --- |
| More neuroanatomy |
| More neuroscience |
| More lectures of clinical neurology |
| More bedside teaching |
| More discussion of cases |
| More online self-learning resources |
| Other comments (please specify) _______________ |

1. **Please choose the specialty that you want to pursue a career in future**

| Cardiology |
| --- |
| Respiratory medicine |
| Gastroenterology |
| Neurology |
| Endocrinology |
| Rheumatology |
| Nephrology |
| Hematology |
| Infectious disease |
| Intensive care unit |
| General practice |
| Geriatrics |
| Not interested in pursuing any of the above (please specify) _______________ |

**Thank you for taking part in this survey. We welcome any other comments you may have on your perception and experience of neurology and how this can be improved.**

___________________________________________________________
